# Comprehensive antitumor immune response boosted by dual inhibition of SUMOylation and MEK in MYC-expressing *KRAS*-mutant cancers

**DOI:** 10.1186/s40164-024-00563-x

**Published:** 2024-09-27

**Authors:** Hiroshi Kotani, Tomoyoshi Yamano, Justin C. Boucher, Shigeki Sato, Hiroyuki Sakaguchi, Koji Fukuda, Akihiro Nishiyama, Kaname Yamashita, Koushiro Ohtsubo, Shinji Takeuchi, Takumi Nishiuchi, Hiroko Oshima, Masanobu Oshima, Marco L. Davila, Seiji Yano

**Affiliations:** 1 Division of Medical Oncology, Cancer Research Institute, Kanazawa University, 13-1 Takara-Machi, Kanazawa, Ishikawa 920-0934 Japan; 2 Department of Immunology, Graduate School of Medical Sciences, Kanazawa University, Kanazawa, Japan; 3 Nano Life Science Institute, Kanazawa University, Kanazawa, Japan; 4 Department of Blood and Marrow Transplant and Cellular Immunotherapy, Division of Clinical Science, H. Lee Moffitt Cancer Center, Tampa, FL USA; 5 Research Center for Experimental Modeling of Human Disease, Kanazawa University, Kanazawa, Japan; 6 Division of Genetics, Cancer Research Institute, Kanazawa University, Kanazawa, Japan; 7 Department of Medicine, Roswell Park Comprehensive Cancer Center, Buffalo, NY USA; 8 Department of Respiratory Medicine, Graduate School of Medical Sciences, Kanazawa University, Kanazawa, Japan

**Keywords:** KRAS, MYC, SUMOylation, TAK-981, Immune response, MEK

## Abstract

Precision medicine has drastically changed cancer treatment strategies including *KRAS*-mutant cancers which have been undruggable for decades. While intrinsic or acquired treatment resistance remains unresolved in many cases, epigenome-targeted therapy may be an option to overcome. We recently discovered the effectiveness of blocking small ubiquitin-like modifier (SUMO) signaling cascade (SUMOylation) in MYC-expressing *KRAS*-mutant cancer cells using a SUMO-activating enzyme E inhibitor TAK-981 that results in SUMOylation inhibition. Interestingly, TAK-981 promoted the degradation of MYC via the ubiquitin–proteasome system. Moreover, combination therapy with TAK-981 and MEK inhibitor trametinib remarkably regressed xenografted *KRAS*-mutant tumors by accumulating DNA damage and inducing apoptosis. Whereas our recent study revealed immune-independent antitumor efficacy, we evaluated the immune responses of cancer cells and immune cells in this study. We found that TAK-981-induced MYC downregulation promoted the activation of STING followed by Stat1 and MHC class I in *KRAS*-mutant cancer cells. Activation of dendritic cells or T cells treated with TAK-981 was also verified by upregulated activation markers in dendritic cells or skew-toward effector-like phenotypes in T cells. Furthermore, the enhanced immune-dependent antitumor efficacy of the combination therapy with TAK-981 and trametinib was confirmed by infiltration of immune cells into tumor tissues and immunodepleting-test using immunodepleting antibodies in syngeneic immunocompetent mouse models. Together with our recent study and here, the findings support that combination inhibition of SUMOylation and MEK comprehensively conquers MYC-expressing *KRAS*-mutant cancers by both immune-dependent and immune-independent antitumor responses.

**Supplementary Information:**

The online version contains supplementary material available at 10.1186/s40164-024-00563-x.

## To the editor,

Precision medicine has drastically changed cancer treatment strategies including *KRAS*-mutant cancer [1]. However, intrinsic or acquired resistance to these therapies remains unresolved. Alternative approaches to overcome these problems may be to target epigenomes [2]. Small ubiquitin-like modifiers (SUMOs) are post-translational modifications (PTMs) that regulate various proteins in many pathways. The conjugation of SUMO to substrate proteins is called SUMOylation, which is caused by an enzymatic cascade consisting of dimeric SUMO-activating enzyme E1 (SAE1/SAE2), a single E2 ubiquitin-conjugating enzyme 9 (UBC9), and a limited set of E3 ligases [3, 4]. The inhibition of SUMOylation is considered a possible treatment option for cancer, and we recently discovered that SUMOylation inhibition using SAE inhibitor TAK-981 induced proteasomal degradation of MYC and effectively suppressed the growth of MYC-expressing *KRAS*-mutant cancers [5]. Although TAK-981 monotherapy showed modest efficacy in vivo*,* combination treatment with TAK-981 and MEK inhibitor trametinib induced drastic antitumor efficacy by DNA damage accumulation and apoptosis in multiple xenograft models.

**Fig. 1:**
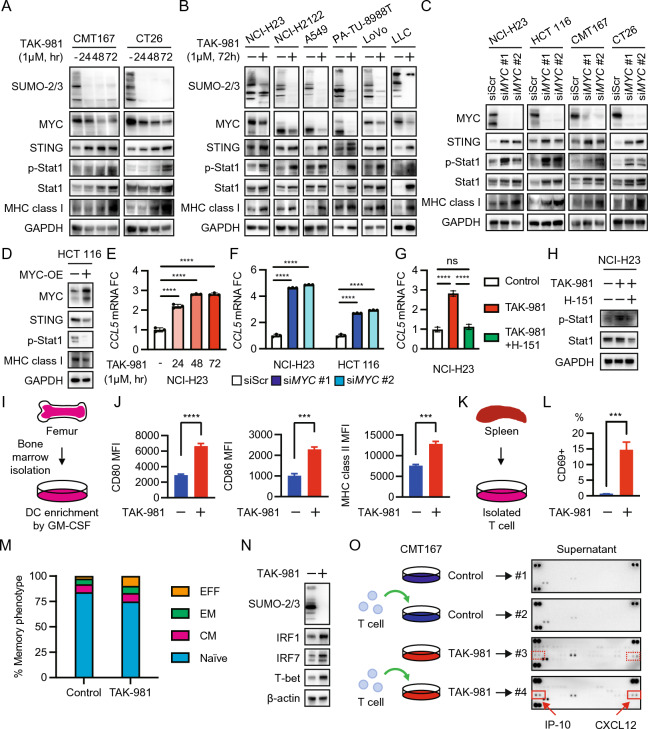
SUMOylation inhibition stimulates antitumor immunological signals via MYC suppression and activates immune cells.** A** Immunoblot of cells treated with DMSO or 1 µM TAK-981 for the indicated period. **B** Immunoblot of cells treated with DMSO or 1 µM TAK-981 for 72 h. **C** Immunoblot of cells transfected with siRNA against MYC or Scr for 3 days. siRNA, short interfering RNA; Scr, scrambled siRNA. **D** Immunoblot of cells transfected with GFP or MYC expressing plasmid. MYC-OE, MYC-overexpression. **E** Fold change in *CCL5* mRNA expression levels in NCI-H23 cells treated with 1 µM TAK-981 for the indicated period. ****p < 0.0001 (Ordinary one-way ANOVA). **F** Fold change in *CCL5* mRNA expression levels in NCI-H23 or HCT 116 cells transfected with siRNA against MYC or Scr for 3 days. ****p < 0.0001 (Ordinary one-way ANOVA). **G,** Fold change in *CCL5* mRNA expression levels in NCI-H23 treated with 1 µM TAK-981 alone or in combination with 1 µM TAK-981 and 1 µM H-151 for 48 h. ****p < 0.0001; ns, not significant (Ordinary one-way ANOVA). **H** Immunoblot of NCI-H23 cells treated with 1 µM TAK-981 in the presence or absence of 1 µM H-151 for 24 h. **I** Overview of DCs’ isolation and enrichment. Mouse bone marrow was isolated from femurs of B6 mice, and DCs were enriched with 20 ng/mL rmGM-CSF for 3 days. B6, C57BL/6 J; DC, dendritic cell; rmGM-CSF, recombinant murine granulocyte–macrophage colony-stimulating factor. **J** Flow cytometry MFIs of the indicated marker on CD11c^+^ DCs cultured in the presence or absence of 1 µM TAK-981 for an additional 3 days after enrichment. ***p < 0.001; ****p < 0.0001 (unpaired t-test). MFI, mean fluorescence intensity. **K,** Overview of T cells’ isolation from mouse spleens. **L, M** Mouse T cell immunophenotyping by flow cytometry. T cells were cultured in the presence or absence of 1 µM TAK-981 for 24 h, immediately after isolation. **L** Percentage of activated T cell phenotype. ***p < 0.001 (unpaired t-test). **M** Percentage of memory T cell phenotypes gated on single/live/CD3^+^. Naïve, CD62L^+^CD44^−^ Naïve-like T cell; CM, CD62L^+^CD44^+^ Central Memory T cell; EM, CD62L^−^CD44^+^ Effector Memory T cell; EFF, CD62L^−^CD44^−^ Effector T cell. **N** Immunoblot of mouse T cells cultured in the presence or absence of 1 µM TAK-981 for 24 h, immediately after isolation. **O,** Immunoblot of mouse cytokine array using the supernatant of 24-h culture following either condition: #1) CMT167 only, #2) CMT167 with isolated T cells at an E:T ratio of 5:1, #3) CMT167 in the presence of 1 µM TAK-981, or #4) CMT167 with isolated T cells at an E:T ratio of 5:1 in the presence of 1 µM TAK-981. 1 × 10^5^ CMT167 cells per well were cultured overnight in a 24-well tissue culture plate. The culture medium was removed, and culture conditions were set up as described above. Finally, the supernatant was collected after 24 h. E:T effector:target

This work investigated how TAK-981 affects immunological signals in MYC-expressing *KRAS*-mutant cancers since TAK-981 has shown potential effects on immune modulation [6–8]. As MYC blocks immune surveillance in various ways [9, 10], we focused on MYC and its downstream signals. In addition, we assessed the effects of TAK-981 on immune cells and the combinatorial effects of TAK-981 and trametinib*.* We first examined immunological signal changes over time and found that MYC downregulation by SUMOylation inhibition activated STING, followed by Stat1 and MHC class I (Fig. 1A). These results were consistent in multiple cell lines (Fig. 1B). In addition, *MYC* knockdown activated the same signals (Fig. 1C), while MYC overexpression suppressed STING expression (Fig. 1D). Furthermore, we assessed the STING response caused by TAK-981-induced MYC downregulation. *CCL5* expression was upregulated by TAK-981 and MYC knockdown (Fig. 1E, F). In addition, *CCL5* upregulation was canceled by a STING antagonist H-151 which blocks the STING-Stat1 axis (Fig. 1G, H). Since *CCL5* upregulation is considered the STING activation effect of a classic Type I interferon response [11], these data indicate that TAK-981-induced MYC downregulation activates STING, which could drive the cancer-immunity cycle [12]. Next, to investigate the effects of TAK-981 on dendritic cells (DCs), we isolated bone marrow cells from B6 mice and enriched DCs with rmGM-CSF (Fig. 1I). DCs treated with TAK-981 expressed higher levels of CD80, CD86, and MHC class II (Fig. 1J, S1), which resulted in activation of innate immunity. We further examined T cells isolated from the spleens of B6 mice (Fig. 1K). We sought to determine the effects of SUMOylation inhibition on T cell immunophenotypes. T cells treated with TAK-981 shifted toward activated CD69^+^ phenotype, and effector-like phenotypes, CD62L^−^CD44^+^ Effector-Memory and CD62L^−^CD44^−^ Effector, compared to the control (Fig. 1L, M, S2). In addition, intracellular signals of IRF-1, IRF-7, and T-bet were upregulated in T cells treated with TAK-981 (Fig. 1N). We also demonstrated mouse cytokine arrays using the supernatant of the culture medium. IP-10 and CXCL12 were secreted at higher levels under TAK-981 treatment during co-culturing with T cells (Fig. 1O). Therefore, these results show that TAK-981 activates T cells in favor of adaptive antitumor immune responses.

**Fig. 2:**
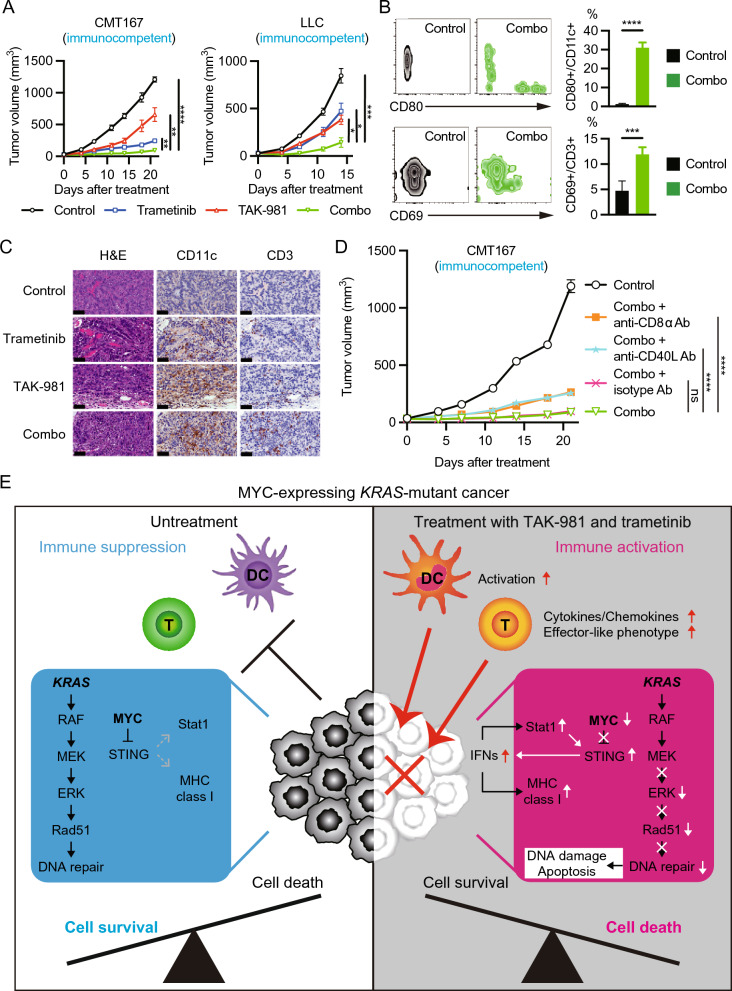
Co-inhibition of SUMOylation and MEK promotes antitumor immune response in MYC-expressing *KRAS*-mutant cancer.** A** Two syngeneic immunocompetent *KRAS*-mutant cancer mouse models using CMT167 or LLC cells treated with vehicle (control), trametinib (0.6 mg/kg, p.o., daily), TAK-981 (25 mg/kg, i.p., twice a week), or both drugs in combination at the same dose. Tumor volumes were plotted over time from treatment initiation (n ≥ 6 per group; mean ± s.e.m.). *p < 0.05; **p < 0.01; ***p < 0.001; ****p < 0.0001 (Ordinary one-way ANOVA). **B** Immune cell subset analysis in peripheral blood of CMT167 tumor graft mouse model treated with vehicle (Control) or combo (trametinib 0.6 mg/kg, p.o., daily and TAK-981 25 mg/kg, i.p., twice a week) for 14 days (n = 5 per group). (Upper) CD80 expression in cells gated on single/live/DUMP-/CD11c + as DCs. (Lower) CD69 expression in cells gated on single/live/DUMP-/CD3 + as T cells. ***p < 0.001; ****p < 0.0001 (unpaired t-test). DUMP, cocktails of TER119, CD11b, Gr1, and NK1.1. **C** Histopathological analyses of CMT167 tumors in B6 mice treated for 7 days as in (**A**). Representative images are shown from three independent experiments. Scale bar, 50 µm. H&E, hematoxylin and eosin. **D** Syngeneic immunocompetent mouse model using CMT167 cells to evaluate the effects on immunodepletion. Mice were treated with vehicle (control), combo (trametinib 0.6 mg/kg, p.o., daily and TAK-981 25 mg/kg, i.p., twice/week), combo plus isotype control IgG2α Ab (10 mg/kg, i.p., once/week), combo plus anti-mouse CD40L Ab (10 mg/kg, i.p., once/week), or combo plus anti-mouse CD8α Ab (10 mg/kg, i.p., once/week). Tumor volumes were plotted over time from treatment initiation (n = 10 per group; mean ± s.e.m.). ****p < 0.0001; ns, not significant (One-way ANOVA). Ab, antibody. **E** Schematic comprehensive mode of action of combination treatment of SUMOylation and MEK inhibition in MYC-expressing *KRAS*-mutant cancer and immune cells. MYC-expressing *KRAS*-mutant cancer cells suppress immune responses of their intra-cellular signals and innate/adaptive immunity. In cancer cells, in addition to induction of DNA damage accumulation and apoptosis by the combination treatment, immunological changes occur such as downregulation of MYC, release of STING suppression, Stat1 activation, and promotion of immunogenicity by the treatment. On the other hand, in immune cells, TAK-981 stimulates DCs to enhance antigen presentation ability and T cells to shift toward effector-like phenotypes and secrete cytokines/chemokines. Collectively, the drug combination cooperatively orchestrates immunotherapeutic effects to drive the Cancer-immunity cycle in addition to immune-independent antitumor effects, which promotes robust antitumor efficacy

To assess the antitumor efficacy of the combination treatment including immune responses in vivo, we performed experiments using two syngeneic immunocompetent mouse models. In addition to xenograft models in our recent report [5], co-inhibition of SUMOylation and MEK effectively suppressed tumor growth in syngeneic models (Fig. 2A, S3). The pharmacodynamic studies also captured immune-dependent and immune-independent effects of combination treatment (Fig. 2B, S4). Moreover, immunohistochemical staining of CMT167 tumor tissue in treated B6 mice revealed that the combination treatment enhanced the infiltration of DCs and T cells in the tumor tissue (Fig. 2C, S5). Finally, we performed an immunodepleting experiment using anti-CD8α antibody (Ab) or anti-CD40L Ab in a CMT167 immunocompetent mouse model. Adding the immunodepleting-Ab to the combination treatment significantly attenuated the efficacy compared to the combination or combination plus isotype control Ab (Fig. 2D).

In conclusion, our data indicate that dual inhibition of SUMOylation and MEK enhances the elimination of MYC-expressing *KRAS*-mutant cancers by modulating the immune response in addition to DNA damage accumulation and apoptosis (Fig. 2E). However, further studies are warranted to elucidate the mechanisms by which immune cells are activated by SUMOylation inhibition.

## Supplementary Information

**Additional file 1. **Materials, Methods, and Abbreviations. **Figure S1.** Gating strategy of DCs and representative histograms of the surface markers. **Figure S2.** Immunophenotypes of mouse T cells. **Figure S3.** Change of individual tumor volume and body weight in mouse models. **Figure S4.** Immunoblotting analysis in pharmacodynamic study.** Figure S5.** Additional histopathological analysis of CMT167 tumors.

## Data Availability

All data supporting the findings of this study are available from the lead contact author upon request. No datasets were generated or analysed during the current study.
