# Supplementary material for: Comprehensive antitumor immune response boosted by dual inhibition of SUMOylation and MEK in MYC-expressing KRAS-mutant cancers
Source: Exp Hematol Oncol. 2024 Sep 27;13:94. doi: 10.1186/s40164-024-00563-x (PMC11438268; doi:10.1186/s40164-024-00563-x)
Supplement: Supplementary file 1 — Additional file 1. Materials, Methods, and Abbreviations. Figure S1. Gating strategy of DCs and representative histograms of the surface markers. Figure S2. Immunophenotypes of mouse T cells. Figure S3. Change of individual tumor volume and body weight in mouse models. Figure S4. Immunoblotting analysis in pharmacodynamic study. Figure S5. Additional histopathological analysis of CMT167 tumors. [file 40164_2024_563_MOESM1_ESM.zip › New folder/Additional file 1_revision.docx]

**Materials, Methods, and Abbreviations**

**Reagents.**

TAK-981, H-151, and trametinib were purchased from Selleck Chemicals. *InVivo*MAb anti-mouse CD8α, *InVivo*MAb anti-mouse CD40L, *InVivo*MAb mouse IgG2α isotype control were purchased from Bio X cell.

**Cells.**

CMT167 cell line was purchased from European Collection of Animal Cell Cultures. LLC cell line was purchased from Japanese Collection of Research Bioresources. CT26 cell line was purchased from Korean Cell Line Bank. A549, HCT 116, LoVo, NCI-H2122, and NCI-H23 cell lines were purchased from American Type Culture Collection. PA-TU-8988T cell line was gifted from the Ebi lab, Aichi Cancer Center. Mouse-derived cell lines were cultured in DMEM supplemented with 10% heat-inactivated fetal bovine serum, 100 U/mL penicillin, and 100 µg/mL streptomycin. Human-derived cell lines were cultured in RPMI 1640 supplemented with 10% heat-inactivated fetal bovine serum, 100 U/mL penicillin, and 100 µg/mL streptomycin. The cells were regularly screened for mycoplasma contamination using a MycoAlert Mycoplasma Detection Kit (Lonza). TexMACS Medium (Miltenyi Biotec) was used for immune cell culture. To enrich for mouse dendritic cells (DCs), cells extracted from the bone marrow of mice were cultured with 20 ng/mL rmGM-CSF (PeproTech). T cells were isolated from the spleens of mice using the EasySep Mouse T cell isolation kit and EasySep Magnet (STEMCELL Technologies). Mouse T cells were not activated by any cytokines or activator beads, such as IL-2 or Dynabeads T-Activator CD3/CD28 throughout the experiment. The cell number and viability were determined using the Countess II FL Automated Cell Counter (Thermo Fisher Scientific). Control cells were treated with 0.1% DMSO throughout the experiment, as described in our previous reports [1,2].

**Immunoblot analysis.**

Cell lysates were collected using CelLytic M (Sigma-Aldrich) supplemented with 1% phosphatase inhibitor cocktail 3 (Sigma-Aldrich) and 10 µM phenylmethanesulfonyl fluoride (Sigma-Aldrich) and the immunodetection of proteins was performed using standard protocols. For the mouse cytokine array, the procedures were performed using the Proteome Profiler Mouse Cytokine Array Kit (R&D Systems) according to the manufacturer’s instructions. Signals were detected using a Chemiluminescence Imaging System (M&S Instruments Inc). The antibodies used are listed below.

| ANTIBODY | SOURCE | IDENTIFIER |
| --- | --- | --- |
| Rabbit monoclonal anti-SUMO-2/3 | Cell Signaling Technology | Cat# 4971, RRID:AB_2198425 |
| Rabbit monoclonal anti-Myc | Cell Signaling Technology | Cat# 13987, RRID:AB_2631168 |
| Rabbit monoclonal anti-STING | Cell Signaling Technology | Cat# 13647, RRID:AB_2732796 |
| Rabbit monoclonal anti-phospho-Stat1 (Tyr701) | Cell Signaling Technology | Cat# 7649, RRID:AB_10950970 |
| Rabbit polyclonal anti-Stat1 | Cell Signaling Technology | Cat# 9172, RRID:AB_2198300 |
| Rat monoclonal anti-MHC class I (Mouse) | Novus | Cat# NB120-15680, RRID:AB_1109327 |
| Mouse monoclonal anti-MHC class I (Human) | Santa Cruz Biotechnology | Cat# sc-32235, RRID:AB_627934 |
| Rabbit monoclonal anti-IRF-1 | Cell Signaling Technology | Cat# 8478, RRID:AB_10949108 |
| Rabbit monoclonal anti-IRF-7 | Cell Signaling Technology | Cat# 72073, D8V1J |
| Rabbit monoclonal anti-T-bet | Cell Signaling Technology | Cat# 97135, E4I2K |
| Rabbit monoclonal anti-phospho-Histone H2A.X (Ser139) | Cell Signaling Technology | Cat# 2577, RRID:AB_2118010 |
| Rabbit monoclonal anti-Cleaved PARP | Cell Signaling Technology | Cat# 94885, RRID:AB_2800237 |
| Rabbit monoclonal anti-GAPDH | Cell Signaling Technology | Cat# 5174, RRID:AB_10622025 |
| Rabbit monoclonal anti-β-actin | Cell Signaling Technology | Cat# 4970, RRID:AB_2223172 |
| Goat polyclonal anti-rabbit IgG, HRP-linked | Cell Signaling Technology | Cat# 7074, RRID:AB_2099233 |
| Goat polyclonal anti-rat IgG, HRP-linked | Cell Signaling Technology | Cat# 7077, RRID:AB_10694715 |
| Horse polyclonal anti-mouse IgG, HRP-linked | Cell Signaling Technology | Cat# 7076, RRID:AB_330924 |

**siRNA.**

Cells were seeded into 60-mm plates at 10% confluency. The next day, the cells were transfected with 40 nM short-interfering RNA (siRNA) against *MYC* (ON-TARGET plus Human/Mouse MYC, Dharmacon) or negative control (Invitrogen) using Lipofectamine RNAiMAX (Invitrogen) according to the manufacturer’s instructions. After three days, the cells were used for subsequent analyses.

**Gene expression analysis.**

RNA was extracted using a RNeasy plus kit (QIAGEN), and cDNA was synthesized using a SuperScript VILO cDNA Synthesis Kit (Invitrogen) according to the manufacturer’s instructions. TaqMan Gene Expression Assays were performed in triplicate for *CCL5* mRNA (Hs00982282_m1, Thermo Fisher Scientific) relative to *GAPDH* mRNA (Hs99999905_m1, Thermo Fisher Scientific) expression. The amount of amplicon was determined using the Mx3005P qPCR System with the TaqMan Universal PCR Master Mix (Applied Biosystems). Data were analyzed using GraphPad Prism 9.

**Flow cytometry.**

Cultured cells were washed twice with PBS and stained with a fixable viability dye. Mouse peripheral blood samples were collected from the facial vein and added RBC lysis buffer according to the manufacturer’s instruction (G-Biosciences). Then, the blood samples were washed thrice with PBS and stained with a fixable viability dye. Surface staining was performed at 4 ºC using an Fc block and a mixture of antibodies in MACS buffer containing 0.5% BSA (Miltenyi Biotec). Samples were analyzed with a 3-laser BD FACSCantoII (BD Biosciences) or 5-laser BD FACSymphony A5 (BD Biosciences), and data were analyzed using FlowJo software (Tree Star). The antibodies used are listed below.

| ANTIBODY | SOURCE | IDENTIFIER |
| --- | --- | --- |
| eFluor 450, Fixable Viability Dye | Thermo Fisher Scientific | Cat# 65-0863 |
| PE-Cy7, anti-mouse CD11c | BD Biosciences | Cat# 558079, RRID:AB_647251 |
| APC, anti-mouse CD11c | BioLegend | Cat# 117310,  RRID: AB_313779 |
| PE, anti-mouse CD80 | BD Biosciences | Cat# 553769, RRID:AB_395039 |
| APC, anti-mouse CD86 | BD Biosciences | Cat# 558703, RRID:AB_2075114 |
| PreCP-Cyanine5.5, anti-mouse I-A/I-E (MHC class II) | BD Biosciences | Cat# 562363, RRID:AB_11153297 |
| FITC, anti-mouse CD3e | Thermo Fisher Scientific | Cat# 11-0031-85, RRID:AB_464883 |
| APC, anti-mouse CD69 | BioLegend | Cat# 104514, RRID:AB_492843 |
| PE/Cyanine7, anti-mouse CD69 | Thermo Fisher Scientific | Cat# 25-0691-82, RRID:AB_469637 |
| PE/Cyanine7, anti-mouse CD62L | BioLegend | Cat# 104418, RRID:AB_313103 |
| APC-eFluor 780, anti-CD44 | Thermo Fisher Scientific | Cat# 47-0441-82, RRID:AB_1272244 |
| eFluor 450, anti-mouse TER-119 | Thermo Fisher Scientific | Cat# 48-5921-82, RRID:AB_158808 |
| eFluor 450, anti-mouse CD11b | Thermo Fisher Scientific | Cat# 48-0112-82, RRID:AB_1582236 |
| eFluor 450, anti-mouse Gr-1 | Thermo Fisher Scientific | Cat# 48-5931-82, RRID:AB_1548788 |
| eFluor 450, anti-mouse NK1.1 | Thermo Fisher Scientific | Cat# 48-5941-82, RRID:AB_2043877 |
| Anti-mouse CD16/32 (FcR blocker) | Thermo Fisher Scientific | Cat# 14-0161-86, RRID:AB_467135 |

**Mice.**

Female 6- to 8-week-old C57BL/6J (B6) (Charles River Laboratories Japan) and CAnN.Cg-*Foxn1^nu^*/CrlCrlj (nude) (Charles River Laboratories Japan) mice were used. The care and treatment of the experimental animals followed the institutional guidelines. 1×10^6^ CMT167 or LLC cells in 100 µL PBS were subcutaneously injected into the flank of B6 on Day -3. On Day 0, treatment with vehicle (control), trametinib (0.6 mg/kg, oral gavage, daily), TAK-981 (25 mg/kg, intraperitoneal injection, twice/week), or both drugs in combination at the same dose was started after randomization. Trametinib was dissolved in equivalent molar concentrations of 7% DMSO, 13% Tween 80, 80% glucose, and 6N HCl equivalent molar concentration to the drug. TAK-981 was dissolved in 20% HPbCD, 2.5% 1N HCl, 2.25% 1N NaOH, and 75.25% deionized water. The mice were monitored daily for body weight and general condition. Tumor volume was measured twice a week using calipers and was calculated using the following formula: length×width^2^×0.5. According to institutional guidelines, mice were sacrificed when their tumor volume reached 1,000 mm^3^.

**Histopathological analysis.** CMT167 tumor tissues treated on B6 mice were collected on Day 7. The tissues were fixed in 10% formalin neutral buffer solution for 2 days. The tissues were then dipped in 70% ethanol and shipped to Morpho Technology. The tissues were appropriately processed according to the manufacturer’s instructions for histopathological analyses, including hematoxylin-eosin, anti-mouse CD3, anti-mouse CD11c, anti-mouse CD8, and phosphorylation of ERK staining. Images were captured using a NanoZoomer (Hamamatsu) and analyzed using NDP.view2 (Hamamatsu).

**Statistical analysis.** The group size was determined based on the preliminary experimental results and no statistical method was used to predetermine the sample size. The indicated sample sizes (n) represent biological replicates. Statistical significance was determined by unpaired two-tailed t-test or Ordinary one-way ANOVA using GraphPad Prism 9. Significance was designated as follows: *p < 0.05; **p < 0.01; ***p < 0.001; ****p < 0.0001; ns, not significant.

**Abbreviations:**

| B6 | C57BL/6J |
| --- | --- |
| CCL5 | chemokine (C-C motif) ligand 5 |
| CRC | colorectal cancer |
| CXCL12 | C-X-C motif chemokine 12 |
| DC | dendritic cell |
| IP-10 | interferon gamma-induced protein 10 |
| IRF | interferon regulatory factor |
| KRAS | v-Ki-ras2 Kirsten rat sarcoma 2 viral oncogene homolog |
| MHC | major histocompatibility complex |
| MYC | v-myc avian myelocytomatosis viral oncogene homolog |
| NSCLC | non-small cell lung cancer |
| PBMC | peripheral blood mononuclear cell |
| PDAC | pancreatic ductal adenocarcinoma |
| PTM | post-translational modification |
| rmGM-CSF | recombinant murine granulocyte-macrophage colony-stimulating factor |
| SAE | small ubiquitin-like modifier-activating enzyme |
| siRNA | short interfering RNA |
| STING | stimulatory interferon genes |
| SUMO | small ubiquitin-like modifier |
| UBC9 | ubiquitin-conjugating enzyme 9 |

**References**

1. Kotani H, Ebi H, Kitai H, Nanjo S, Kita K, Huynh TG, et al. Co-active receptor tyrosine kinases mitigate the effect of FGFR inhibitors in FGFR1-amplified lung cancers with low FGFR1 protein expression. Oncogene. 2016;35(27):3587–97. <https://www.nature.com/articles/onc2015426>

2. Kotani H, Adachi Y, Kitai H, Tomida S, Bando H, Faber AC, et al. Distinct dependencies on receptor tyrosine kinases in the regulation of MAPK signaling between BRAF V600E and non-V600E mutant lung cancers. Oncogene. 2018;37(13):1775–87. <https://www.nature.com/articles/s41388-017-0035-9>
